# Exogenous Melatonin Improves Seed Germination of Wheat (*Triticum aestivum* L.) under Salt Stress

**DOI:** 10.3390/ijms23158436

**Published:** 2022-07-29

**Authors:** Jiajie Wang, Penghui Lv, Di Yan, Zhendong Zhang, Xiaomeng Xu, Ting Wang, Ye Wang, Zhen Peng, Chunxin Yu, Yuerong Gao, Liusheng Duan, Runzhi Li

**Affiliations:** Beijing Key Laboratory of New Technology in Agricultural Application, National Experimental Teaching Demonstration Center for Plant Production, Beijing University of Agriculture, Beijing 102206, China; jiajie.wang@bua.edu.cn (J.W.); penghui.lv@bua.edu.cn (P.L.); di.yan@bua.edu.cn (D.Y.); zhendong.zhang@bua.edu.cn (Z.Z.); xiaomeng.xu@bua.edu.cn (X.X.); ting.wang@bua.edu.cn (T.W.); ye.wang@bua.edu.cn (Y.W.); zhen.peng@bua.edu.cn (Z.P.); yuchunxin@cau.edu.cn (C.Y.); yuerong.gao@bua.edu.cn (Y.G.)

**Keywords:** salt stress, melatonin, seed germination, hormone, temporal transcriptome

## Abstract

Melatonin (MT) can effectively reduce oxidative damage induced by abiotic stresses such as salt in plants. However, the effects of MT on physiological responses and molecular regulation during wheat germination remains largely elusive. In this study, the response of wheat seeds to MT under salt stress during germination was investigated at physiological and transcriptome levels. Our results revealed that application of MT significantly reduced the negative influence of salt stress on wheat seed germination. The oxidative load was reduced by inducing high activities of antioxidant enzymes. In parallel, the content of gibberellin A3 (GA_3_) and jasmonic acid (JA) increased in MT-treated seedling. RNA-seq analysis demonstrated that MT alters oxidoreductase activity and phytohormone-dependent signal transduction pathways under salt stress. Weighted correlation network analysis (WGCNA) revealed that MT participates in enhanced energy metabolism and protected seeds via maintained cell morphology under salt stress during wheat seed germination. Our findings provide a conceptual basis of the MT-mediated regulatory mechanism in plant adaptation to salt stress, and identify the potential candidate genes for salt-tolerant wheat molecular breeding.

## 1. Introduction

Salt stress is becoming an important limiting factor for crop growth, development and productivity [1], which causes osmotic, ionic and oxidative stress to plants [2,3,4]. Up to now, more than 20% of arable land (approximately 10 million hectares) has been affected by salinization, and this number is still increasing as consequence of global climate change and arable land mismanagement [5,6]. Osmotic stress reduces the water absorption of the plants and ionic stress caused by the excessive accumulation of Na^+^ increases the ionic toxicity resulting in the inhibition of photosynthesis, impairment of ion homeostasis, and peroxidation of membrane lipids, which further inhibit plants growth under salt stress [7]. The accumulation of reactive oxygen species (ROS) induced by salt stress can cause membrane lipid peroxidation and finally reduce the photosynthesis in plants [8,9]. Seed germination is the initial and important stage of early plant growth, which is vulnerable and sensitive to abiotic and biotic stress [10]. Due to the excessive accumulation of Na^+^ under salt stress, the plasma membrane permeability and structure of seed cell is altered and eventually leading to oxidative damage to seeds [11].

To adapt to the salt stress, plants evolved a series of defense systems to prevent the damage during seed germination, including regulating the endogenous hormone levels, improving the activity of the antioxidant enzymes, and activating the salt overly sensitive (SOS) pathway [12,13,14]. Among these defense systems, ROS scavenging, mainly mediated by antioxidant enzymes, is an important pathway for improving the abiotic stress tolerance of plants, such as peroxidase (POD), superoxide dismutase (SOD) and catalase (CAT). Increasing the activity of antioxidant enzymes can effectively improve the ability to scavenge ROS, reduce plasma membrane damage, and improve tolerance to abiotic stresses [8,9]. For example, overexpression of SOD and CAT genes protects transgenic plants from oxidative damage, and enhances tolerance to abiotic stress [15,16]. In addition, maintaining ion homeostasis is an effective strategy in response to salt stress by regulating the ion transport systems [17]. Overexpression of SOS genes and plasma membrane Na^+^ and K^+^ transporters genes can maintain ion homeostasis, and significantly improve salt tolerance in plants [18,19,20,21]. These results revealed that that maintaining Na^+^/K^+^ homeostasis and reducing the ionic toxicity caused by excessive accumulation of Na^+^ can further improve the salt tolerance in plants [2].

Plant hormones, as central regulators in plant developmental and defense processes [22], can activate stress responsive genes to enhance plant stress tolerance. Abscisic acid (ABA) plays a significant role in plant response to abiotic stresses and participates in maintenance of cellular osmotic potential. In addition, the crosstalk of ABA with other phytohormone-mediated signal pathways can further help plants to adapt to extreme environments [23,24]. Also, application of exogenous ABA can significantly improve plant stress tolerance [25,26], which further confirmed the importance of ABA in plant response to abiotic stresses. ABA and GA are the main endogenous factors regulating seed dormancy and germination. ABA/GA balance is the core regulatory mechanism that determines the maintenance or change of seed dormancy [27,28]. GA plays an important role in promoting seed germination, stalk elongation, fruit development and improving plant stress tolerance [29]. Application of exogenous GA can protect plants from salt stress and improve the salt tolerance of plants [30,31]. In addition, salt stress can induce an increase in JA levels in plants [32]. Application of exogenous methyl jasmonate (JA-Me) induces protection against oxidative stress and increases activities of the antioxidant enzymes under salt stress [32]. These results revealed that plant hormones play an important function in the regulation of plant response to abiotic stress.

Melatonin (N-acetyl-5-methoxytryptamine, MT), as an indoleamine-type hormone, is considered a new hormone related to seed germination and response to environmental stress [33]. MT can protect the plant to avoid stress-induced damage via reducing the accumulation of ROS and improving the antioxidant enzyme activity [34]. It was showed that exogenous MT improves the antioxidant enzyme activity and maintains the osmotic pressure in tomato plants [35], and enhances cotton leaf sugar metabolism under drought stress [36]. Synergistic effects of MT and SA on modulating ion homeostasis relieve the damage induced by salt stress in wheat [37,38]. Seeds treated with MT can promote seed germination by improving the antioxidant system and starch metabolism under chilling stress [39,40]. However, the molecular mechanisms of MT-mediated regulations of seed germination and salt stress tolerance remain unclear.

Bread wheat (*Triticum aestivum* L.), a vital food crop in the world, is threatened by various abiotic stresses during the whole growth process [41,42]. Therefore, how to improve wheat stress tolerance has become a research hotspot. Previous studies showed that MT improves seed germination [33], but it has been less applied to wheat under salt stress. Our study shows that application of MT significantly improves wheat seed germination under salt stress. Although the studies about the effect of MT on plant stress tolerance are gradually increasing, MT-mediated physiological responses and molecular regulation mechanism during wheat germination still need to be further explored. To investigate the effects of exogenous application of MT on the antioxidant enzyme system and endogenous hormones in wheat seeds, we measured the antioxidant enzyme activities and endogenous hormone contents. Our RNA-seq analysis revealed that MT participates in enhanced energy metabolism and protects the wheat seeds via maintained cell morphology under salt stress during seed germination.

## 2. Results

### 2.1. The Influence of Exogenous MT on Wheat Seed Germination under Salt Stress

To investigate the effects of MT on wheat germination under salt stress, we assessed the germination rate (GR), germination potential (GP), germination index (GI), and vigor index (VI) of wheat seeds. Compared with the water control (CK), all indexes of salt-treated groups decreased significantly; whereas indexes of MT-treated groups were significantly increased compared with the salt control (S). It indicates that salt stress could effectively inhibit wheat seed germination, and application of MT significantly alleviated the effect of salt injury on wheat seed germination (Table 1). According to the investigation of the seed germination, the GR of the SM4 group significantly increased by 10.67% compared with S. GP, GI and VI significantly increased by 10%, 9.14% and 197.77, respectively. The trend of increase was more significant than compared with other SM groups (Table 1). The results revealed that application of MT could significantly relieve the inhibition effects of salt stress on wheat seed germination.

### 2.2. The Influence of Exogenous MT on SOD and CAT Activities and MDA Content of Wheat Seedlings under Salt Stress

**Figure 1:**
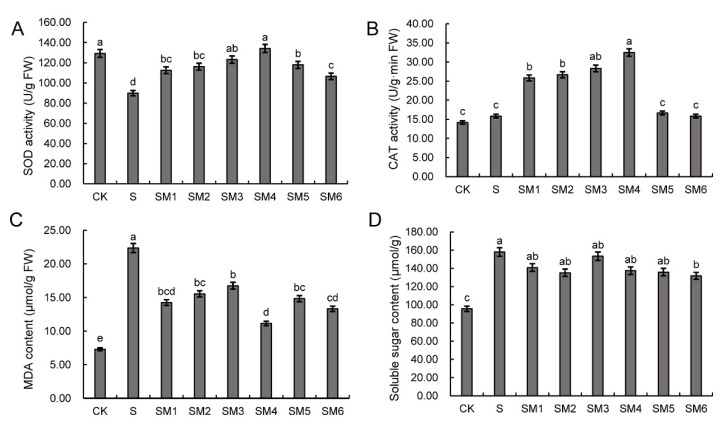
Changes in physiology in wheat seedlings under water control (CK), salt stress (S), and salt stress with different MT concentrations (SM) treatment conditions. (**A**–**D**) Changes in the SOD activates (**A**), CAT activities (**B**), MDA contents (**C**), and soluble sugar contents (**D**). The error bar in the figure represents the standard deviation (SD, *n* = 3). Different letters indicate significant differences by the Duncan test (*p* < 0.05).

To further analyze the effects of MT on wheat seedlings under salt stress, we investigate physiological indexes of wheat seedlings under different treatment conditions. The activities of the SOD and CAT of wheat seedlings under SM treatments were significantly higher than that of S treatment. The activities of SOD and CAT of wheat seedlings reached a maximum at SM4 treatment (Figure 1A,B). Under salt stress, the MDA content of wheat seedlings under S treatment (22.37 μmol/g) was significantly higher than those of other treatments (Figure 1C). The MDA content of wheat seedlings in SM groups was generally significantly lower than that in the S treatment. The lowest MDA content of wheat seedlings was observed in the SM4 group, compared with other groups (Figure 1C). Meanwhile, the soluble sugar contents of the S and SM groups were significantly increased compared with CK, but there was no significant difference between the S and SM groups (Figure 1D). This result further revealed that application of MT could significantly relieve the inhibition effects of salt stress on wheat seed germination and seedling growth.

### 2.3. The Influence of Exogenous MT on Endogenous Hormones of Wheat Seed under Salt Stress

**Figure 2:**
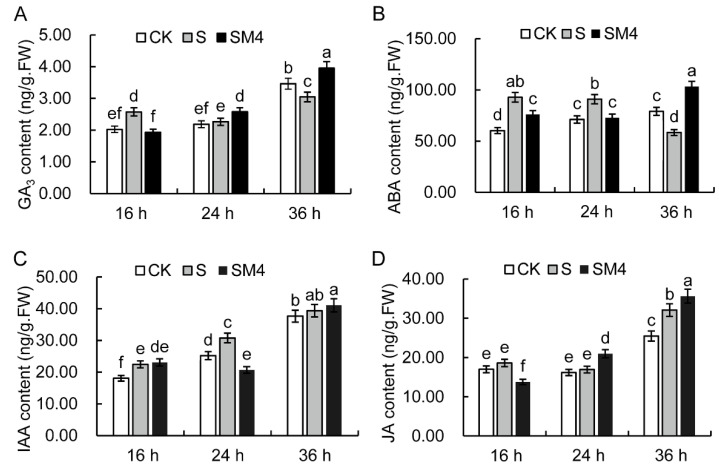
Changes in different hormones in wheat seedlings under water control (CK), salt stress (S), and salt stress with 200 μM MT (SM4) treatment at different time points (16 h, 24 h, 36 h). (**A**–**D**) Changes in the content of GA_3_ (**A**), ABA (**B**), IAA (**C**), and JA (**D**). The error bar in the figure represents the standard deviation (SD, *n* = 3). Different letters indicate significant differences by the Duncan test (*p* < 0.05).

Endogenous hormones are vital during the seed germination process [14,43], and the change levels of several plant hormones were investigated by liquid chromatography with mass spectrometry (LS-MS). The results showed that four plant hormones, including GA_3_, ABA, auxin, and JA, were increased during seed germination under the normal condition (Figure 2). Compare with CK, the content of GA_3_ was significantly increased at 16 h, and those of ABA and auxin (IAA) was increased at 16 and 24 h under salt stress (Figure 2A–C), and the content of GA_3_ and ABA after salt stress treated at 36 h was markedly lower than that of on the CK condition (Figure 2A,B). Compared with salt stress, the contents of GA_3_, ABA and JA were decreased at 16 h and significantly increased at 36 h by application of MT (Figure 2A,B,D). These results revealed that application of MT could affect the levels of plant hormones during wheat seed germination.

### 2.4. The Influence of Exogenous MT on Functional Gene Expressions of Wheat Seed under Salt Stress

**Figure 3:**
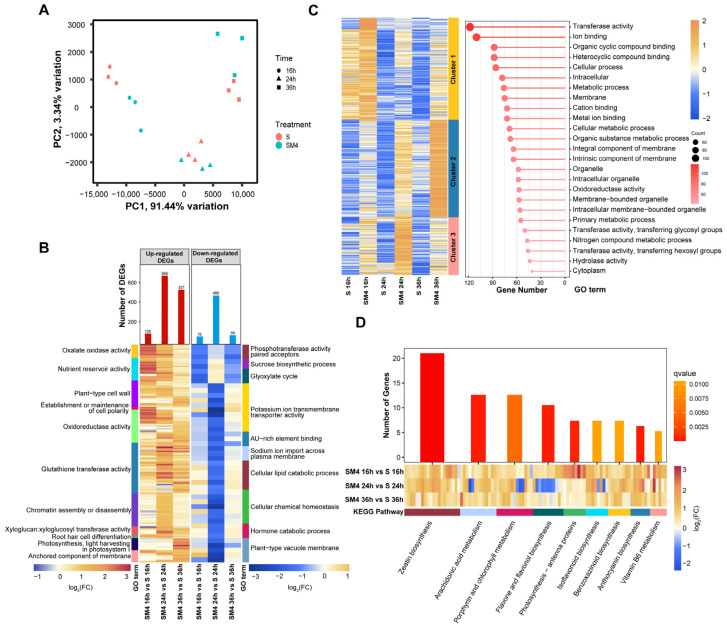
Overview of the transcriptomic changes during three different time samples of S and SM4. (**A**) Principal component analysis (PCA) of all samples. (**B**) Gene Ontology (GO) enrichment analysis of deferentially expressed genes (DEGs) in three-time points. The total number of up-regulated DEGs (red) or down-regulated DEGs (blue) in each comparison was shown above each bar. Heatmap of DEGs in the GO terms (q-value < 0.05) that were affected by MT. (**C**) The heatmap of three clusters in which the genes were up-regulated in SM4 condition, and the GO annotation of the DEGs in these three clusters. (**D**) Kyoto Encyclopedia of Genes and Genomes (KEGG) pathway analysis of all DEGs. The color of the bar chart showed the significance levels (q-value), and the color scale of the heat map represents up-regulated (red) and down-regulated (blue).

To explore the molecular mechanism of MT-induced wheat salt tolerance, we performed RNA-seq under different treatments (S and SM4). Over 10 Gb raw data per sample abstained with the Q30 of SM4 treatment and S treatment were more than 89% and the clean reads ratio accounted from 80.27% to 91.95% (Table S1). The expression patterns of five selected genes for qRT-PCR analysis were consistent with the RNA-seq data (Figure S1), demonstrating that RNA-seq data were dependable and appropriate for further analysis. To provide an overview to understand how wheat genes are regulated to the response of the S and SM4 treatment at different time points, the principal component analysis (PCA) of all transcriptomes globally was carried out (Figure 3A). Transcriptomes of 16, 24, and 36 h and the model of S and SM4 transcriptome compartmentalization revealed significant transcriptional variation between S and SM4 at different time points (Figure 3A).

Compared with salt treatment (S), 181 deferentially expressed genes (DEGs) (105 up-regulated and 76 down-regulated), 1312 DEGs (666 up-regulated and 466 down-regulated), and 616 DEGs (527 up-regulated and 89 down-regulated) were respectively identified at SM4 treatment for 16 h, 24 h, and 36 h (Figure 3B). GO enrichment analysis of DEGs showed that the GO terms related to “oxalate oxidase activity”, “oxidoreductase activity”, “glutathione transferase activity”, and “establishment or maintenance of cell polarity” were enriched under MT and salt stress treatment conditions. The down-regulated DEGs were enriched in the GO terms related to “potassium ion transmembrane transporter activity”, “sodium ion import across plasma membrane”, “cellular lipid catabolic process”, “cellular chemical homeostasis” and “hormone catabolic process” (Figure 3B). 12 clusters were classified according to the DEGs expression patterns (Figure S2), and clusters 1, 2 and 3, which consist of 388 DEGs, were mainly up-regulated by MT at different time points (Figure 3C, Table S2), while these DEGs were related to the oxidoreductase activity, transferase activity, hydrolase activity and cellular metabolic process according to GO annotation (Figure 3C).

To further investigate the effect of MT on the biosynthesis and metabolism pathway, the DEGs were enriched using KEGG pathway analysis, and the results found that the genes involved in “zeatin biosynthesis”, “photosynthesis”, “flavone and flavonol biosynthesis”, “anthocyanin biosynthesis” and “vitamin B6 metabolism” pathways were significantly induced by MT under salt stress (Figure 3D). In addition, there were 24 DEGs enriched in the pathway of “plant hormone signal transduction” (Table S3). *PYR/PYL*, *SnRK2* and *ABF* in the ABA respond pathway were up-regulated, the same as *AUX/IAA* in the IAA pathway and *GID1* in the GA pathway, but *DELLA* in the GA pathway was down-regulated at 24 h (Figure S3). These results suggested that MT protected the wheat seedlings under salt stress via maintained cell morphology, adjusted osmotic potential, and increased the antioxidative enzyme activities and the biosynthesis of antioxidants.

### 2.5. Weighted Gene Co-Expression Network Analysis (WGCNA)

**Figure 4:**
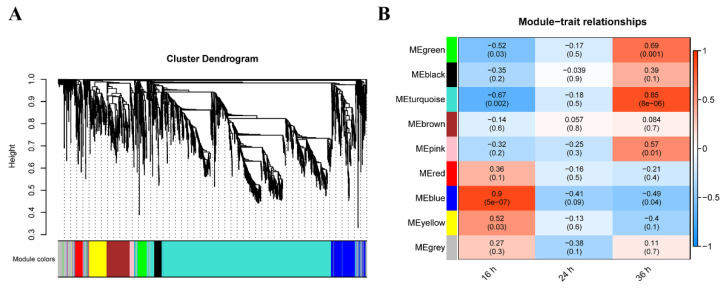
Weighted gene co-expression network analysis of differentially expressed genes. (**A**) Hierarchical clustering tree of DEGs by WGCNA. (**B**) Module–time relationships. The value above the box represents the correlation coefficient between the module and time, and the value below represents the significance between them.

WGCNA was used to examine the key genes and functionally enriched pathways in the effected of MT under salt stress. It was shown that all the DEGs were clustered into nine modules (Figure 4A). The MEblue module was significantly positively correlated with 16 h (*r* = 0.9, *p* = 5 × 10^−7^), but negatively correlated with 36 h (*r* = −0.49, *p* = 0.04), whereas the MEgreen (*r* = 0.69, *p* = 0.001), MEturquoise (*r* = 0.85, *p* = 8 × 10^−6^) and MEpink (*r* = 0.57, *p* = 0.01) module were significantly positively correlated with 36 h (Figure 4B).

**Figure 5:**
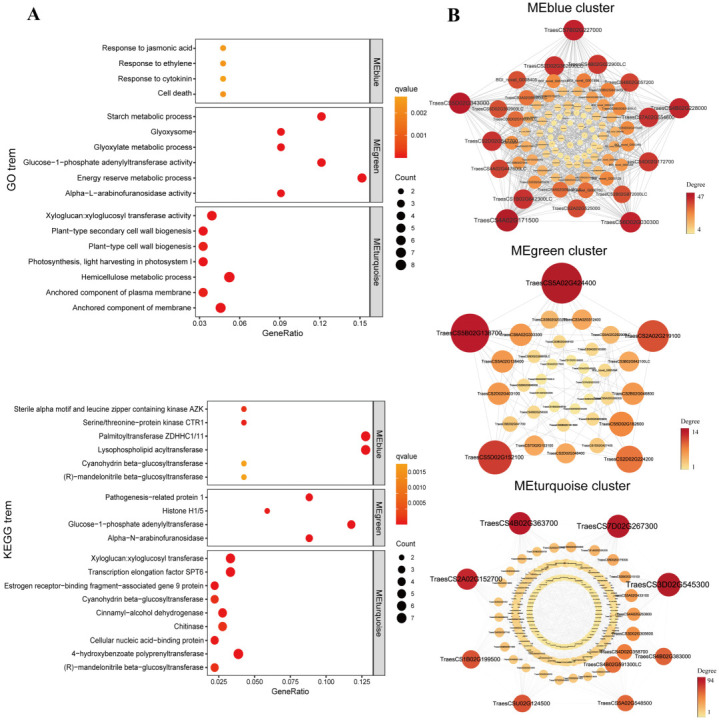
Analysis of module MEblue, MEgreen, and MEturquoise. (**A**) The GO enrichment and KEGG Orthologs analysis. The bubble size indicated the number of genes enriched in the GO term (or KEGG Orthologs), and the color represented the degree of significance. (**B**) The co-expression network of DEGs in MEblue, MEgreen, and MEturquoise modules. Degree indicated the number of lines connected to the node, and each line connected two different genes.

GO analysis and KEGG analysis were performed on three modules (MEblue, MEgreen and MEturquoise) significantly associated with 16 and 36 h (Tables S4 and S5). GO analysis showed that the genes enriched in the MEblue module correlated with hormone response, such as “response to ethylene”, “response to jasmonic acid” and “response to cytokinin” (Figure 5A), which indicated that MT altered the plant hormone signal transduction of wheat early germination stage under salt stress. In the MEgreen module, the “energy reserve metabolic process”, “glyoxylate metabolic process”, “glucose-1-phosphate adenylyltransferase” and “starch metabolic process” were enriched according to GO and KEGG pathway enrichment analysis (Figure 5A), suggested the MT may participate in enhanced energy metabolism in seed germination. In the MEturquoise module, the genes were enriched in “xyloglucan: xyloglucosyl transferase activity”, “anchored component of membrane”, “photosynthesis, light harvesting in photosystem I”, and “cellular nucleic acid-binding protein” (Figure 5A) indicated that MT protect the wheat seedlings via maintained cell morphology under salt stress during seed germination.

To screen and identified hub genes, the DEGs with the highest degree in these three modules were selected for co-expression gene network mapping (Figure 5B). A total of 18 highly weighted hub genes were identified (5 in the MEblue module, 5 in the MEgreen module and 8 in the MEturquoise module, Table S6). Of them, 6 genes may be vital for MT to promote seed germination under salt stress (Table S6), including *TraesCS4B02G228000* (*EDR7*, EARLY-RESPONSIVE TO DEHYDRATION 7, chloroplastic-like) and *TraesCS1B02G199500* (*SVB5*), which orthologues in *Arabidopsis thaliana* were involved in the response of ABA to salt stress [44,45,46]. *TraesCS2A02G152700* (*MYB46*), *TraesCS5A02G548500* (*XTH31*, XYLOGLUCAN ENDO-TRANSGLYCOSYLASE/HYDROLASE 31-like) and *TraesCS4B02G383000* (*XTH31*) were associated with cell wall formation [47,48]. *TraesCS7D02G267300* (PTI1-3, PTI1-like tyrosine-protein kinase 3) may be activated by oxidative stress [49]. These hub genes are considered as candidators to further investigate their role in the response to salt stress.

## 3. Discussion

### 3.1. MT Improved the Germination Rate of Wheat by Increasing the Activities of Antioxidant Enzymes under Salt Stress

Seed germination period was the initial plant growth, which was important for seed emergence rate. Salt stress reduces seed germination, damages the internal cells of seeds, and leads to growth retardation in a variety of crops [50,51,52,53]. Wheat seed germination was sensitive to salt stress [54]. In this study, we found that salt stress inhibited the germination of wheat seeds, and increased the content of MDA in wheat seedlings (Table 1, Figure 1C). Previous studies showed that application of MT can promote seed germination under abiotic stress [55,56,57,58]. Our study further demonstrates that low concentrations of MT can promote seed germination and high concentration of MT inhibit seed germination under salt stress.

Antioxidant enzymes play a role in scavenging ROS and their activities increase when plants are suffering from salt stresses [59]. MT was reported to enhance the activities of POD, CAT and SOD, eliminate ROS, reduce cell damage and modify the gene expression of antioxidant enzymes in previous studies [60,61,62,63,64]. Our study showed that exogenous MT increased the germination rate by increasing activities of antioxidant enzymes (CAT and SOD) under salt stress (Table 1, Figure 1). The MDA content generated by ROS-induced lipid membrane damage [65] was significantly lower in seeds treated with MT compared to seeds without treatment under salt stress (Figure 1). It is noteworthy that there was no significant difference between the soluble sugar content of the S group and the SM group, but both were significantly higher than that of the CK group. Although MT can improve salt tolerance in wheat, wheat cells still need to be protected from osmotic stress by maintaining a high concentration of soluble sugars. These results suggest that MT can increase antioxidant enzyme activity at the physiological level and protect the plasma membrane from oxidative damage.

Glutathione S-transferase (GST) can improve plant tolerance to salt stress [66,67]. Application of exogenous MT, which induced up-regulation of GST-related gene *GLCAT14B* [68]. Exogenous MT also up-regulated the expression of GST genes in kiwifruit seedlings and wheat seedlings, respectively [69,70]. In our study, a total of 15 genes related to GST activity were significantly up-regulated by treatment with MT under salt stress, and the up-regulation was more significant at 24 h (Figure S4). The results of transcriptome analysis showed that MT attenuates salt stress-induced cytotoxicity by regulating the ascorbate-glutathione cycle. These results further confirm that increasing the activity of antioxidant enzymes and reducing ROS damage are the main ways for MT to improve salt tolerance in plants.

### 3.2. MT Modified the Phytohormones Responses of Wheat Early Germination Stage under Salt Stress

Plant hormones mediate the regulation of biotic and abiotic stress responses [71,72,73]. ABA is involved in responses to abiotic stresses and is demonstrated to be induction and maintenance of seed dormancy, while IAA, GA, and JA can induce seed germination and promote radicle or germ growth [74,75,76,77]. Previous studies showed that MT improves plant salt tolerance and seed germination mainly by regulating the levels of ABA and GA [57,78,79]. Our results showed the contents of GA_3_ and JA increased significantly by MT treatment under salt stress. we found that MT also promotes seed germination and growth by affecting the level of JA.

In this study, the DEGs involved in plant hormone signal transduction and hormone response were identified according to GO enrichment analysis and WGCNA (Figure 5 and Figure S3, Table S3). The expression of genes related to both ABA and GA signaling pathways was altered in cucumber and cotton seeds after melatonin treatment under salt stress [57,78]. *PYR/PYL*, *SnRK2*, and *ABF* are important components of the ABA signaling pathway [80,81]. In this study, *PYR/PYL* was induced by MT, which further inhibited *PP2C* and upregulated *SnRK2* and *ABF* expression, thereby increasing ABA levels in response to salt stress (Figure S3). Perhaps there were other unknown substances or pathways involved in the regulation, resulting in a significant increase in ABA content only after seed germination (at 36 h). In addition, the *GID1* and *DELLA* in the GA signaling pathway are also regulated by MT. GID1 (a GA receptor) interacts with DELLA protein (a repressor of GA signaling) in a GA-dependent manner [82]. Our results showed that MT significantly upregulated *GID1* expression and down-regulated *DELLA* expression during germination, which increased GA levels and promoted seed germination (Figure S3). These results indicated that MT improved wheat salt stress tolerance via modified plant hormone signal transduction and affects the synthesis and metabolism of the phytohormones.

### 3.3. MT Affects Multiple Molecular Mechanisms to Promote Seed Germination under Salt Stress

High salt concentration induces ionic stress in plants, which adversely affects plant growth. Excessive Na^+^ and Cl^−^ flow into plant cells results in ion toxicity and imbalance [83,84]. Plants have developed defense systems to maintain ionic balance by regulating the Na^+^ and K^+^ levels in the cytoplasm [85]. In this study, the DEGs related to Na^+^ import across the plasma membrane and K^+^ transmembrane transporter activity were identified to respond to MT treatment under salt stress (Figure 3B). MT can regulate gene expression of ion transporters on membranes to maintain intracellular Na^+^/K^+^ homeostasis; however, the molecular mechanism of MT-mediated regulation of Na^+^/K^+^ homeostasis was still unclear. 

The KEGG pathway enrichment analysis showed that MT could enhance the zeatin biosynthesis pathway (Figure 3D). Cytokinin is considered associated with plant development and salt stress response [86,87,88,89]. In grape berries and kiwifruits, the flavonoid content was increased after melatonin treatment and the related genes involved in flavonoids and anthocyanins were significantly up-regulated [90,91]. Flavonoids and anthocyanin participate in the growth and development of plant organs and involve in the response to abiotic stress [92,93,94,95,96,97]. The gene involved in flavonoids and anthocyanin biosynthesis were up-regulated by MT treatment to enhance antioxidant capacity under salt stress, which implies that MT can play an important role in further regulating the antioxidant system by regulating the synthesis and metabolism of a variety of substances. 

Taken together, MT promoted the salt tolerance of wheat seeds via regulating the expression pattern of genes associated with flavonoids biosynthesis, phytohormone biosynthesis, signal transduction, and antioxidant system. It provides the theoretical basis of the MT mediated-regulatory mechanism in plant adaptation to salt stress.

### 3.4. A Model of the Molecular Mechanism of MT Promoted Seed Germination under Salt Stress

**Figure 6:**
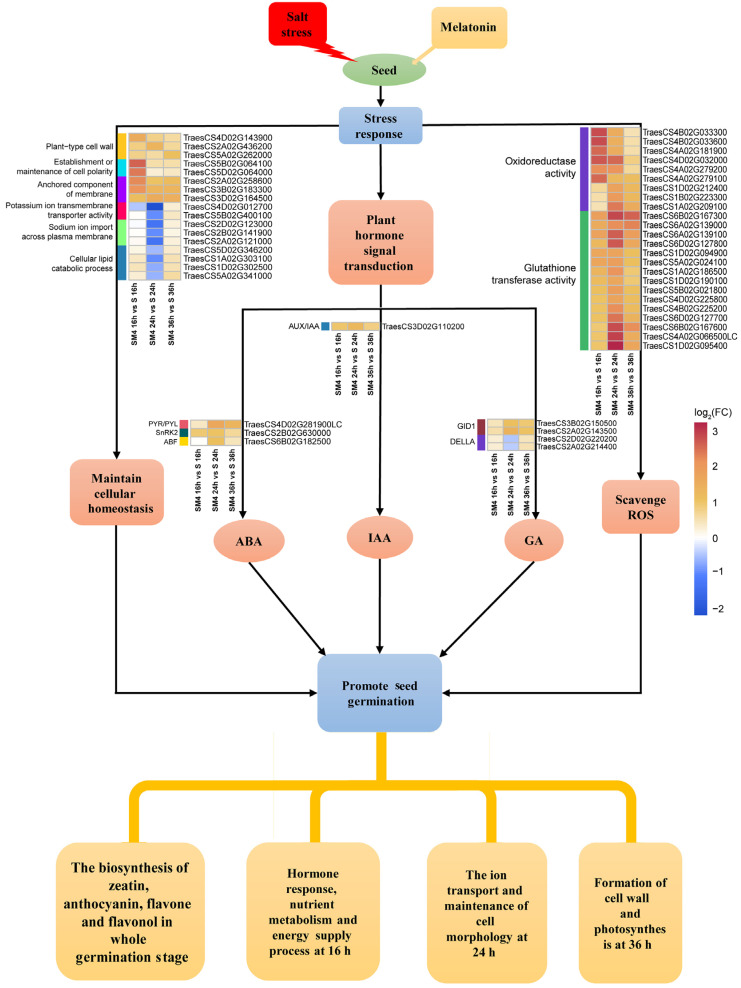
Schematic diagram of a model for MT to promote seed germination. MT maintained the cellular homeostasis of seeds, scavenged ROS, and improved the salt tolerance of seeds through the co-regulation of hormones signal transduction, flavonol biosynthesis, and antioxidant systems.

A model for MT induced high wheat seed germination under salt stress was proposed (Figure 6). At the early stage of germination (16 h) of wheat seed under salt stress, the application of MT affected the plant hormone response and enhanced nutrient metabolism and energy supply process. Exogenous MT is involved in regulating the balance between ABA and GA to promote seed germination. Exogenous MT affected the synthesis and metabolic pathways of IAA and zeatin, and alleviated the developmental retardation caused by salt stress. When the seeds grew radicles (24 h), exogenous MT regulated genes related to the ion transport pathway to maintain intracellular ion homeostasis and promote seed development. In addition, exogenous MT regulated genes involved in maintaining cellular morphology to improve seed vigor under salt stress. Exogenous MT promotes cell wall formation and affects photosynthesis when the seeds grow germ (36 h), which alleviates the slow growth and development. The biosynthesis pathway of zeatin, anthocyanin, flavone and flavonol, the hormone signaling pathways, and the abilities of scavenging ROS were enhanced under the influence of MT, resulting in improving the tolerance of wheat seeds to salt stress and promote seed germination.

## 4. Materials and Methods

### 4.1. Reagents

MT was purchased from Bio Basic Inc. (BBI, Shanghai, China) and other chemicals were purchased from Sinopharm Chemical Reagent Beijing Co., Ltd. (Beijing, China). All chemicals used in the experiments were analytical grade.

### 4.2. Plant Material and Germination Tests

Bread wheat (cv. Jimai22) was used in this study, and wheat seeds were germinated in the germination box after being sterilized with 1% sodium hypochlorite in an incubator (SaFe, Ningbo, China) with 16 h photoperiods (25 °C/20 °C, day/night temperature) and relative humidity of approximately 60%. To explore the relationship between salt stress response and MT, seven salt stress treatments were set: 200 mM NaCl (selected in the pre-experiment, Figure S5) was added with 0 µM, 50 µM, 100 µM, 150 µM, 200 µM, 250 µM, and 300 µM MT (S, SM1, SM2, SM3, SM4, SM5 and SM6), respectively, and the seeds germinated in distilled water were served as the control (CK). Each treatment was performed in three biological replicates.

### 4.3. Morphological Observation

The characteristic of seed germination is that the germinated root tip breaks through the seed coat and the visible radicle appears. The germination potential and germination rate of seeds were counted on the 3rd and 7th day, respectively, and the seedling height on the 7th day was measured.

Germination rate (GR) = (total number of germinated grains/total number of grains) × 100%

Germination potential (GP) = (number of germinated seeds on the 3rd day/total number of test seeds) × 100%

Germination index (GI) = Σ (Gt/Dt)

where Gt is the total number of seeds germinated per day, and Dt is the corresponding germination days.

Mean germination time (MGT) = Σ (Gt × Dt)/ΣGt

Vigor index (VI) = S × GI

S refers to the seedling height that was measured on the 7th.

### 4.4. Measurement of Antioxidant Enzyme Activities, MDA, and Soluble Sugar Content 

After 7 days treated, the leaves of seedlings were obtained to detect the content of malondialdehyde (MDA) and soluble sugar according to the method described by Diao [98]. The activities of superoxide dismutase (SOD) and catalase (CAT) were measured following the previously described [99,100]. All measurements were performed in three biological replicates.

The crude extracts of wheat leaves were extracted as follows: put the wheat material in a precooled mortar and quickly grind it to powder with liquid nitrogen. Add 0.2 g powder to a 2 mL centrifuge tube to determine antioxidant enzyme activities and add 0.2 g powder to a 10 mL centrifuge tube to determine MDA and soluble sugar content. The 2 mL centrifuge added pH 7.8 sodium phosphate buffer, then centrifuged in a centrifuge (4 °C, 12,000× *g*, 20 min) and collected the supernatant for enzyme activity assay. All steps of the extraction procedure were carried out at 4 °C.

The content of MDA and soluble sugar were detected by the thiobarbituric acid (TBA) method [98]. Briefly, 8 mL 10% trichloroacetic acid was added to a 10 mL centrifuge tube containing 0.2 g of homogenized wheat leaves and centrifuged in a centrifuge (4 °C, 12,000× *g*, 10 min). A volume of 2 mL TBA was added to the 2 mL supernatant in a water bath at 95 °C for 20 min. Then centrifuge again, collect the supernatant, and measure the absorbance at 450, 532, and 600 nm, respectively.

The SOD activity was detected by nitrogen blue tetrazolium (NBT). Briefly, 100 μL of the extracted supernatant was taken and 3.9 mL of the reaction mixture prepared with NBT was added. The mixture was measured at 560 nm after being exposed to light at 4000 Lx for 30 min. The CAT activity was estimated by measuring the decrease rate of H_2_O_2_. The H_2_O_2_ was added to the enzyme extract mixed with buffer and measured at 240 nm for 1 min.

### 4.5. Extraction and Assay of Phytohormones

The endogenous hormone contents were determined by taking wheat seeds from groups S and SM4 at 16 h, 24 h, and 36 h of germination, respectively. All samples were immediately frozen in liquid nitrogen and stored at −80 °C. The sample was mixed with methanol/water/formic acid (15:4:1, V/V/V). The mixture was vibrated for 10 min and then centrifuged at 12,000 r/min and 4 °C for 5 min. The supernatant was evaporated and dissolved in 80% methanol (V/V), and then filtered through a membrane filter for further LC–MS.

The content of IAA, GA_3_, JA, and ABA of wheat seeds were measured by Wuhan Metware Biotechnology Co. Ltd. (Wuhan, China) based on the AB Sciex QTRAP4500 LC-MS platform. Each assay contained three biological replicates.

### 4.6. RNA-seq and Weighted Correlation Network Analysis (WGCNA)

Total RNA was isolated from the wheat seeds that were treated with S and SM4 for 16 h, 24 h, and 36 h using the Plant Total RNA Kit (Beijing Zoman, Beijing, China) according to the manufacturer’s protocol. Each treatment group at different time points contained three biological replicates. The transcriptome sequencing was performed by Beijing Genomics Institute (Beijing, China) based on the MGISEQ-2000 platform. The clean reads were compared to the genome sequence by Bowtie2 (v2.2.5, Ben Langmead, Baltimore, MD, USA) [101], and then RSEM (v1.2.8, Bo Li, Madison, WI, USA) [102] was used to calculate the gene expression of each sample. DESeq2 (v1.34.0, Michael I Love, Boston, Germany) [103] was utilized for identifying the DEGs (deferentially expressed genes) between SM treatments and S treatments, and clusterProfiler (v4.2.2, Guangchuang Yu, Guangzhou, China) [104] was used for GO and KEGG pathways analysis. The clustering of gene expression patterns was carried out using Mfuzz (v2.54.0, Lokesh Kumar, Berlin, Germany) [105] in R.

Weighted correlation network analysis package in R (WGCNA, v1.70-3, Peter Langfelder, Los Angeles, CA, USA) [106] was used to analyze all the DEGs. To describe gene association patterns between distinct samples, we used a soft threshold to form an undirected network. In order to produce suitable gene sets, the minimum module size was set to 30, and the minimum merged height was set to 0.25. The gene co-expression network was visualized and hub genes were identified via the Cytoscape software (v3.9.0, Institute for Systems Biology, Washington, DC, USA).

### 4.7. Quantitative Real-Time PCR (qRT-PCR) Analysis

Gene-specific primers were designed by Primer Premier 5.0 software (Primer Premier, Ottawa, ON, Canada) and listed in Table S7, and qRT-PCR was carried out on an Applied Biosystems 7500 real-time PCR system using SYBR Green Premix Pro Taq HS Kit II (Accurate Biotechnology, Changsha, China) following the manufacturer’s protocol. The relative expression level was calculated by the 2^−∆∆CT^ method [107], which used the expression of *Actin1* and *Actin2* to be the standers [108]. The samples used correspond to the RNA-seq and contain three biological replicates for each of the different treatments at different time points.

### 4.8. Statistical Analysis

One-way ANOVA, which was utilized for evaluating the significant difference and final results were expressed as mean ± standard error, was carried out by IBM SPSS Statistics 22 software (IBM Corp, Armonk, NY, USA), and *p* < 0.05 was considered statistically significant.

## Figures and Tables

**Table 1:** Effect of different treatments on the wheat seed germination and seedling height.

Treatment	GR (%)	GP (%)	GI	VI	MGT (d)	Seedling Height (cm)
CK	100.00 ± 0.00 a	78.00 ± 5.29 a	44.11 ± 1.59 a	704.64 ± 17.78 a	1.27 ± 0.09 e	15.98 ± 0.23 a
S	72.00 ± 2.00 f	63.33 ± 4.00 d	17.26 ± 1.07 g	73.23 ± 8.84 g	2.18 ± 0.13 a	4.25 ± 0.49 g
SM1	76.67 ± 1.15 e	73.67 ± 1.15 b	19.00 ± 0.22 f	97.70 ± 2.66 f	2.05 ± 0.04 b	5.14 ± 0.12 f
SM2	80.00 ± 2.00 d	68.00 ± 2.67 c	21.09 ± 0.93 e	125.06 ± 9.58 e	2.03 ± 0.05 b	5.92 ± 0.19 de
SM3	82.67 ± 1.15 d	74.67 ± 1.15 b	21.54 ± 0.40 de	169.04 ± 10.05 c	2.00 ± 0.04 bc	7.85 ± 0.54 c
SM4	92.67 ± 1.15 b	73.33 ± 4.00 b	26.67 ± 0.87 b	271.00 ± 18.47 b	1.90 ± 0.02 cd	10.16 ± 0.54 b
SM5	86.00 ± 2.00 c	70.00 ± 2.00 c	22.67 ± 0.38 d	147.71 ± 2.69 d	2.05 ± 0.07 b	6.52 ± 0.23 d
SM6	80.67 ± 2.31 d	64.67 ± 2.67 d	24.39 ± 0.34 c	137.89 ± 8.20 de	1.83 ± 0.06 d	5.65 ± 0.30 ef

Values are mean ± standard deviation (SD, *n* = 3). Different letters indicate significant differences by using the Duncan test (*p* < 0.05). GR, germination rate; GP, germination potential; GI, germination index; VI, vigor index; MGT, mean germination days, CK, water control; S, salt stress without MT; SM1, salt stress with 50 μM MT; SM2, salt stress with 100 μM MT; SM3, salt stress with 150 μM MT; SM4, salt stress with 200 μM MT; SM5, salt stress with 250 μM MT; SM6, salt stress with 300 μM MT.

## Data Availability

The RNA-seq dataset in this study has been uploaded to SRA database in NCBI (BioProject ID: PRJNA783016).

## Supplementary Materials

The following supporting information can be downloaded at: https://www.mdpi.com/article/10.3390/ijms23158436/s1.
