# Supplementary material for: Exogenous Melatonin Improves Seed Germination of Wheat (Triticum aestivum L.) under Salt Stress
Source: Int J Mol Sci. 2022 Jul 29;23(15):8436. doi: 10.3390/ijms23158436 (PMC9368970; doi:10.3390/ijms23158436)
Supplement: Supplementary file 1 [file ijms-23-08436-s001.zip › ijms-1824929-supplementary.pdf]

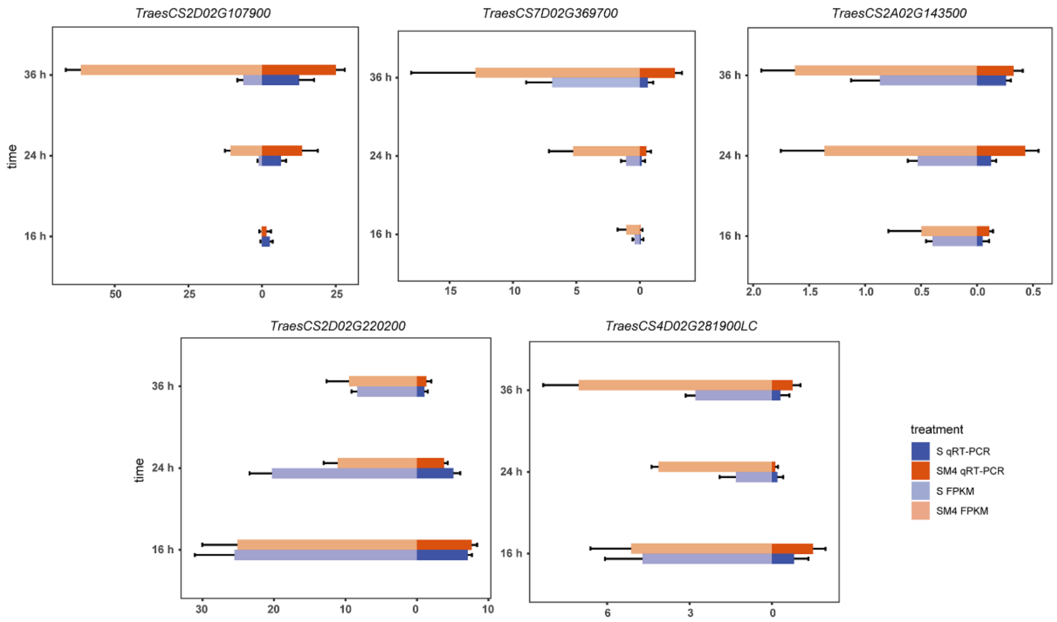

Figure S1. Effects of melatonin on gene expression under salt stress in wheat seed by qRT-PCR. Genes: TraesCS2D02G107900, TraesCS7D02G369700, TraesCS2A02G143500, TraesCS2D02G220200, and TraesCS4D02G281900LC. SM4: germination of seeds treated with 200 μM melatonin under 200 mM NaCl solution; S: germination of seeds treated with 200 mM NaCl.

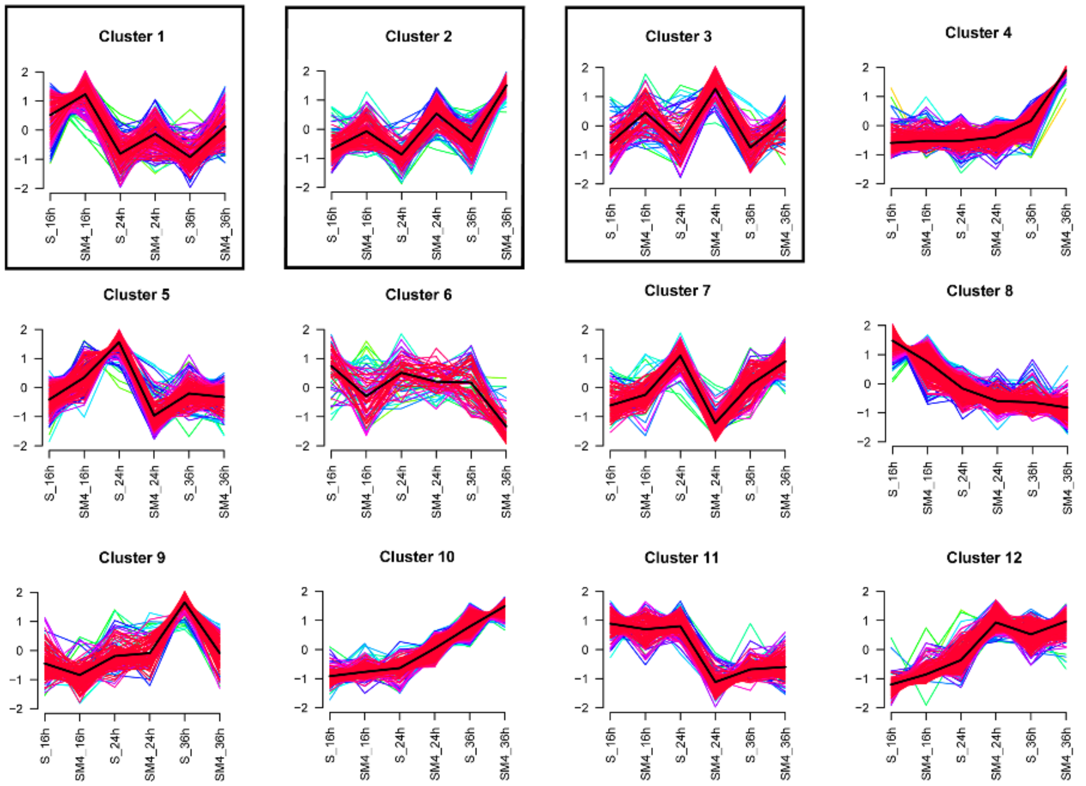

Figure S2. Clustering of all DEGs based on their expression patterns in S and SM4 at 16 h, 24 h and 36 h. 12 clusters shown different expression patterns, and cluster 1, cluster 2 and cluster 3 were selected to further analysis. The abscissa represented different treatment in different time and ordinate represented normalized expression value. The black lines represented the mean expression trend of DEGs (other lines) belonging to each cluster.

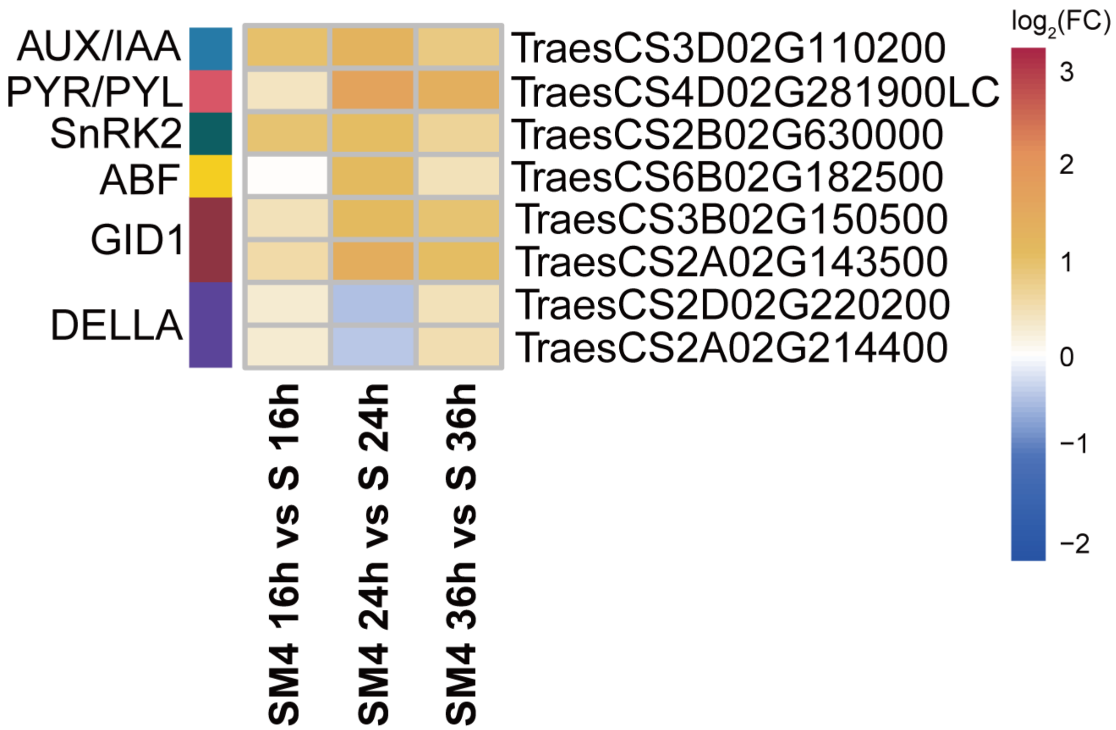

Figure S3. The heatmap of the DEGs in the pathway of plant hormone signal transduction. The color scale of the heat map represents up-regulated (red) and down-regulated (blue).

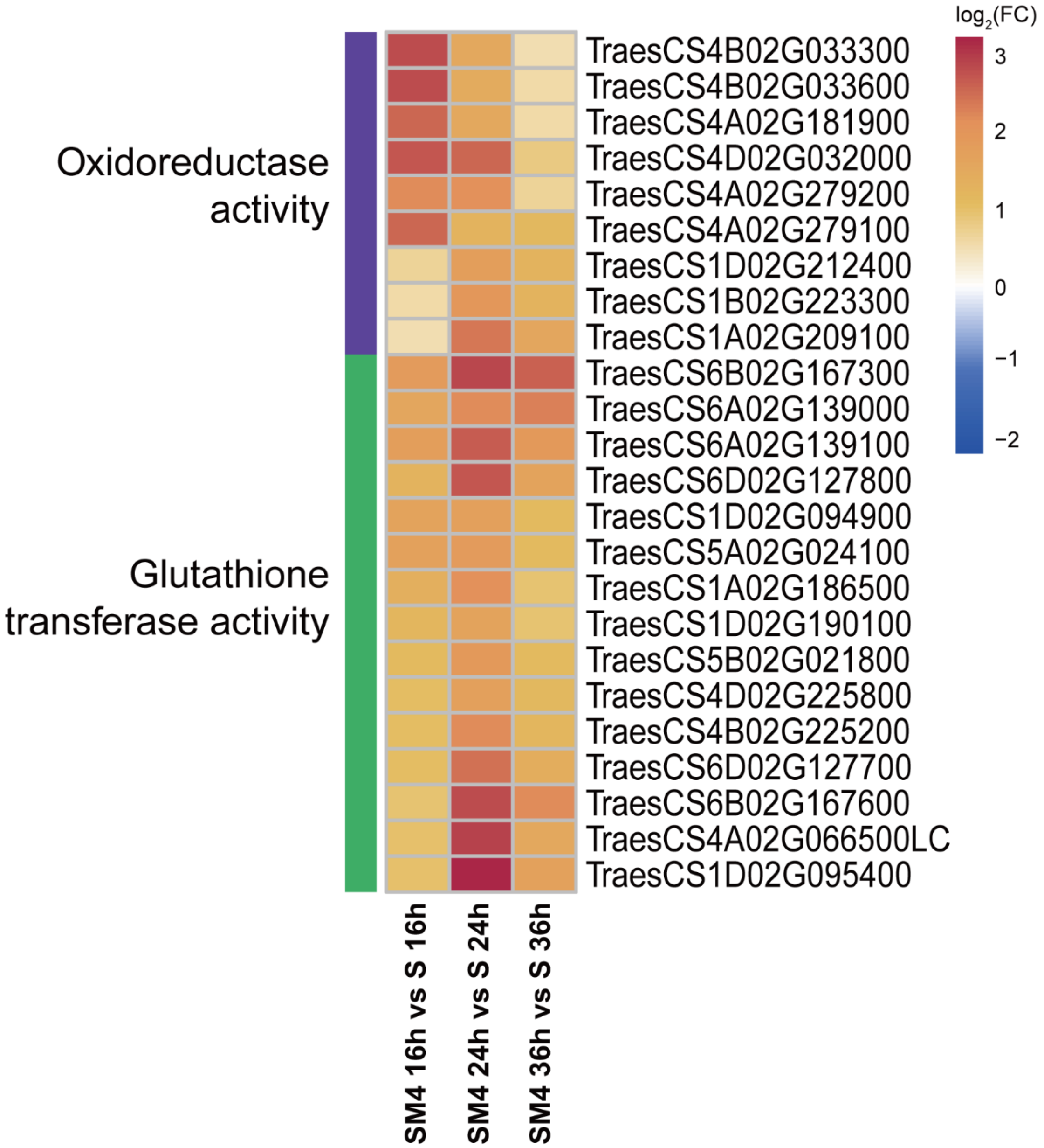

Figure S4. The heatmap of the DEGs in oxidoreductase activity and glutathione transferase activity. The color scale of the heat map represents up-regulated (red) and down-regulated (blue).

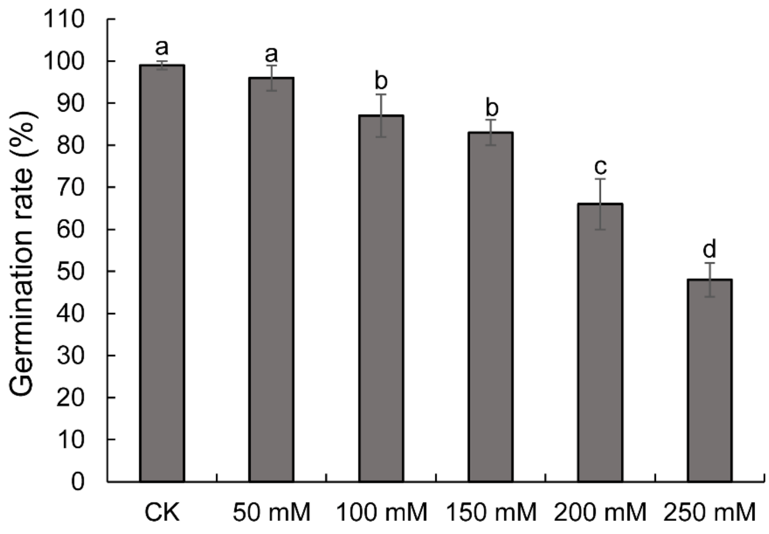

Figure S5. The germination rate of the wheat seeds in different NaCl concentration. The error bar in the figure represents the SD (n = 3). Different letters represent significant ($P < 0.05$).

Table S1. Quality statistics of filtered transcriptome Reads.

Sample	Total Raw Reads (M)	Total Clean Reads (M)	Total Clean Bases (Gb)	Clean Reads Q20 (%)	Clean Reads Q30 (%)	Clean Reads Ratio (%)
SM4-16h-1	82.7	69.01	10.35	95.29	89.44	83.44
SM4-16h-2	86.01	69.26	10.39	95.36	89.61	80.52
SM4-16h-3	82.7	69.28	10.39	95.39	89.58	83.77
SM4-24h-1	79.4	67.88	10.18	95.59	90.03	85.5
SM4-24h-2	82.7	68.77	10.32	95.45	89.66	83.15
SM4-24h-3	82.7	69.82	10.47	95.53	89.91	84.43
SM4-36h-1	82.7	68.73	10.31	95.75	90.38	83.1
SM4-36h-2	79.4	67.26	10.09	95.3	89.42	84.72
SM4-36h-3	73.62	67.69	10.15	95.82	90.16	91.95
S-16h-1	79.4	67.21	10.08	95.41	89.64	84.66
S-16h-2	79.4	67.34	10.1	95.58	90.02	84.81
S-16h-3	82.7	68.72	10.31	95.47	89.8	83.09
S-24h-1	79.4	67.67	10.15	95.45	89.72	85.23
S-24h-2	82.7	69.71	10.46	95.37	89.53	84.29
S-24h-3	82.7	69.58	10.44	95.45	89.74	84.13
S-36h-1	82.7	68.96	10.34	95.44	89.69	83.38
S-36h-2	86.01	69.04	10.36	95.43	89.78	80.27
S-36h-3	82.7	68.16	10.22	95.48	89.8	82.41

Table S2. The DEGs number in the clusters of the DEGs expression patterns.

Cluster	Number	Cluster	Number	Cluster	Number
Cluster 1	154	Cluster 2	147	Cluster 3	87
Cluster 4	512	Cluster 5	160	Cluster 6	68
Cluster 7	131	Cluster 8	286	Cluster 9	112
Cluster 10	528	Cluster 11	153	Cluster 12	300

Table S3. DEGs in plant hormone signal transduction.

Gene ID	Pathway Name	Level 1	Level 2
TraesCS2A02G143500	ko04075//Plant hormone signal transduction	Environmental Information Processing	Signal transduction
TraesCS2A02G214400	ko04075//Plant hormone signal transduction	Environmental Information Processing	Signal transduction
TraesCS2A02G433500	ko04075//Plant hormone signal transduction	Environmental Information Processing	Signal transduction
TraesCS2B02G630000	ko04016//MAPK signaling pathway - plant;ko04075//Plant hormone signal transduction	Environmental Information Processing	Signal transduction
TraesCS2D02G220200	ko04075//Plant hormone signal transduction	Environmental Information Processing	Signal transduction
TraesCS2D02G437500LC	ko04075//Plant hormone signal transduction;ko04016//MAPK signaling pathway - plant	Environmental Information Processing	Signal transduction
TraesCS3B02G150500	ko04075//Plant hormone signal transduction	Environmental Information Processing	Signal transduction
TraesCS3B02G537400	ko04075//Plant hormone signal transduction	Environmental Information Processing	Signal transduction
TraesCS3B02G882200LC	ko01212//Fatty acid metabolism;ko01040//Biosynthesis of unsaturated fatty acids;ko00061//Fatty acid biosynthesis;ko04075//Plant hormone signal transduction	Metabolism; Environmental Information Processing	Global and overview maps; Signal transduction; Lipid metabolism
TraesCS3D02G110200	ko04075//Plant hormone signal transduction	Environmental Information Processing	Signal transduction
TraesCS3D02G429400	ko04075//Plant hormone signal transduction	Environmental Information Processing	Signal transduction
TraesCS4B02G012100	ko03015//mRNA surveillance pathway;ko04016//MAPK signaling pathway - plant;ko04075//Plant hormone signal transduction	Environmental Information Processing; Genetic Information Processing	Translation; Signal transduction
TraesCS4D02G281900LC	ko04016//MAPK signaling pathway - plant;ko04075//Plant hormone signal transduction	Environmental Information Processing	Signal transduction
TraesCS5A02G183300	ko04626//Plant-pathogen interaction;ko04016//MAPK signaling pathway - plant;ko04075//Plant hormone	Environmental Information Processing; Organismal Systems	Signal transduction; Environmental adaptation

	signal transduction		
TraesCS5B02G181500	ko04016//MAPK signaling pathway - plant;ko04075//Plant hormone signal transduction;ko04626//Plant-pathogen interaction	Environmental Information Processing; Organismal Systems	Environmental adaptation; Signal transduction
TraesCS5D02G259800LC	ko04075//Plant hormone signal transduction;ko04626//Plant-pathogen interaction;ko04016//MAPK signaling pathway - plant	Environmental Information Processing; Organismal Systems	Signal transduction; Environmental adaptation
TraesCS6B02G182500	ko04075//Plant hormone signal transduction	Environmental Information Processing	Signal transduction
TraesCS7A02G328100	ko04075//Plant hormone signal transduction	Environmental Information Processing	Signal transduction
TraesCS7A02G461700	ko04075//Plant hormone signal transduction	Environmental Information Processing	Signal transduction
TraesCS7B02G049000	ko04075//Plant hormone signal transduction	Environmental Information Processing	Signal transduction
TraesCS7B02G228900	ko04075//Plant hormone signal transduction	Environmental Information Processing	Signal transduction
TraesCS7B02G335000	ko04075//Plant hormone signal transduction	Environmental Information Processing	Signal transduction
TraesCS7D02G370700	ko04075//Plant hormone signal transduction	Environmental Information Processing	Signal transduction
TraesCS7D02G426000	ko04075//Plant hormone signal transduction	Environmental Information Processing	Signal transduction

Table S4. The most enriched GO terms of different modules.

	ID	Description	p.adjust	Count
MEblue in 16 h	GO:0010286	heat acclimation	0.000677	2
	GO:0008219	cell death	0.007547	2
	GO:0009723	response to ethylene	0.007547	2
	GO:0009753	response to jasmonic acid	0.008019	2
	GO:0009735	response to cytokinin	0.00862	2
MEgreen in 36 h	GO:0008878	glucose-1-phosphate adenylyltransferase activity	1.70E-10	4
	GO:0006112	energy reserve metabolic process	4.95E-10	5
	GO:0006097	glyoxylate cycle	2.23E-08	3
	GO:0046487	glyoxylate metabolic process	2.23E-08	3
	GO:0019252	starch biosynthetic process	4.10E-08	4
	GO:0070566	adenylyltransferase activity	4.30E-08	4
	GO:0019566	arabinose metabolic process	5.52E-08	3
	GO:0046373	L-arabinose metabolic process	5.52E-08	3
	GO:0009514	glyoxysome	5.96E-08	3
	GO:0005982	starch metabolic process	1.35E-07	4
	GO:0046556	alpha-L-arabinofuranosidase activity	6.40E-07	3
	GO:0016833	oxo-acid-lyase activity	1.64E-06	3
	GO:0019321	pentose metabolic process	2.35E-06	3
	GO:0005777	peroxisome	6.86E-06	4
	GO:0042579	microbody	6.86E-06	4
	GO:0006099	tricarboxylic acid cycle	4.19E-05	3
	GO:0006101	citrate metabolic process	4.36E-05	3
	GO:0006081	cellular aldehyde metabolic process	7.28E-05	3
MEturquoise in 36 h	GO:0009768	photosynthesis, light harvesting in photosystem I	2.05E-07	5
	GO:0010410	hemicellulose metabolic process	1.31E-06	8
	GO:0016762	xyloglucan:xyloglucosyl transferase activity	1.31E-06	6
	GO:0031225	anchored component of membrane	3.06E-06	7
	GO:0009834	plant-type secondary cell wall biogenesis	3.51E-06	5
	GO:0010411	xyloglucan metabolic process	3.74E-06	6
	GO:0009765	photosynthesis, light harvesting	7.85E-06	5
	GO:0008171	O-methyltransferase activity	1.90E-05	6
	GO:0010287	plastoglobule	2.05E-05	5
	GO:0071949	FAD binding	7.97E-05	6
	GO:0009832	plant-type cell wall biogenesis	0.000165	5

GO:0046658	anchored component of plasma membrane	0.000168	5
GO:0046271	phenylpropanoid catabolic process	0.000168	4
GO:0046274	lignin catabolic process	0.000168	4
GO:0052716	hydroquinone:oxygen oxidoreductase activity	0.000168	4
GO:0009698	phenylpropanoid metabolic process	0.000169	5
GO:0009522	photosystem I	0.000392	5
GO:0009808	lignin metabolic process	0.000392	4
GO:0032578	aleurone grain membrane	0.000498	2
GO:0033095	aleurone grain	0.000498	2

Table S5. The KEGG Orthology of different modules.

	ID	Description	p.adjust	Count
MEblue in 16 h	K20027	palmitoyltransferase ZDHHC1/11	1.11E-10	6
	K13519	lysophospholipid acyltransferase	3.07E-10	6
	K14510	serine/threonine-protein kinase CTR1	0.000317	2
	K04424	sterile alpha motif and leucine zipper containing kinase AZK	0.000768	2
	K13030	cyanohydrin beta-glucosyltransferase	0.007638	2
	K22706	(R)-mandelonitrile beta-glucosyltransferase	0.007638	2
MEgreen in 36 h	K00975	glucose-1-phosphate adenylyltransferase	3.30E-09	4
	K01209	alpha-N-arabinofuranosidase	5.20E-08	3
	K13449	pathogenesis-related protein 1	5.45E-06	3
	K11275	histone H1/5	0.000627	2
	K14423	methylsterol monooxygenase 1	0.008588	1
	K16298	serine carboxypeptidase-like clade IV	0.008588	1
	K01006	pyruvate, orthophosphate dikinase	0.008588	1
MEturquoise in 36 h	K06125	4-hydroxybenzoate polyprenyltransferase	1.02E-12	7
	K11292	transcription elongation factor SPT6	7.41E-09	6
	K22455	estrogen receptor-binding fragment-associated gene 9 protein	4.53E-06	4
	K08235	xyloglucan:xyloglucosyl transferase	6.18E-06	6
	K22395	cinnamyl-alcohol dehydrogenase	5.15E-05	5
	K09250	cellular nucleic acid-binding protein	5.64E-05	4
	K20495	long-chain fatty acid omega-monooxygenase	0.000205	3
	K14641	apyrase	0.000335	3
	K13030	cyanohydrin beta-glucosyltransferase	0.000383	4
	K22706	(R)-mandelonitrile beta-glucosyltransferase	0.000423	4
	K01183	chitinase	0.000436	5
	K05681	ATP-binding cassette, subfamily G (WHITE), member 2	0.000578	4
	K17506	protein phosphatase 1L	0.002597	3
	K01568	pyruvate decarboxylase	0.003087	4
	K01870	isoleucyl-tRNA synthetase	0.003659	3

Table S6. Hub genes statistics of different modules.

Module	Gene ID	Degree	Annotation
MEblue	TraesCS4A02G171500	47	PREDICTED: RGS domain-containing serine/threonine-protein kinase A-like.[Triticum aestivum]
	TraesCS5D02G343000	44	PREDICTED: serine/threonine-protein kinase STY46-like.[Triticum aestivum]
	TraesCS6D02G030300	42	PREDICTED: lysine-specific demethylase JMJ25-like. [Triticum aestivum]
	TraesCS7B02G227000	42	PREDICTED: tubby-like F-box protein 12. [Triticum aestivum]
	TraesCS4B02G228000	41	PREDICTED: protein EARLY-RESPONSIVE TO DEHYDRATION 7, chloroplastic-like. [Triticum aestivum]
MEgreen	TraesCS5A02G424400	14	PREDICTED: peroxisomal (S)-2-hydroxy-acid oxidase GLO1-like. [Triticum aestivum]
	TraesCS5B02G138700	13	PREDICTED: alpha-L-arabinofuranosidase 1-like. [Triticum aestivum]
	TraesCS5D02G152100	11	PREDICTED: alpha-L-arabinofuranosidase 1-like. [Triticum aestivum]
	TraesCS2A02G219100	10	TaSAG7 mRNA for isocitrate lyase. [Triticum aestivum]
	TraesCS2D02G224200	8	PREDICTED: isocitrate lyase. [Triticum aestivum]
MEturquoise	TraesCS3D02G545300	94	PREDICTED: putrescine hydroxycinnamoyltransferase 3-like. [Triticum aestivum]
	TraesCS7D02G267300	91	PREDICTED: PTI1-like tyrosine-protein kinase 3. [Triticum aestivum]
	TraesCS4B02G363700	88	PREDICTED: probable apyrase 3. [Triticum aestivum]
	TraesCS2A02G152700	81	PREDICTED: transcription factor MYB46-like. [Triticum aestivum]
	TraesCSU02G124500	66	PREDICTED: 7-deoxyloganetin glucosyltransferase-like. [Triticum aestivum]
	TraesCS1B02G199500	66	PREDICTED: uncharacterized protein. [Triticum aestivum]
	TraesCS4B02G383000	61	PREDICTED: xyloglucan endotransglucosylase/hydrolase protein 31-like. [Triticum aestivum]
	TraesCS5A02G548500	59	PREDICTED: xyloglucan endotransglucosylase/hydrolase protein 31-like. [Triticum aestivum]

Table S7. Primers designed by qRT-PCR.

gene name	Primer sequences (5′-3′)
TraesCS2D02G107900	F: ATCGGATCTACGGCGGTGAC<br>R: CGAAGTTGCGGACGGTGTT
TraesCS7D02G369700	F: TCATGGCTGCCGCTGTCT<br>R: CGGTGAAGTTGGCGGTGT
TraesCS2A02G143500	F: CGACGAAGAACCAAGAAACAGG<br>R: TCGCCGAAGGAGAAGAGGAA
TraesCS2D02G220200	F: ATGGCACCTGATGCTGAT<br>R: ACTCCCTTTGTCGTTTCT
TraesCS4D02G281900LC	F: CCTTCTCGGCGGTCTTGG<br>R: GCACCGGCTCAAGAACTACCT
Actin1 (As the internal reference gene)	F: CACCGCCGAACGGGAAAT<br>R: AAGGACCTCAGGGCAACG
Actin2 (As the internal reference gene)	F: CCAGTACTGCTGACTGAGGC<br>R: TGTTGTGCGTCCACTAGCAT
